# Paracentesis in Hydrothorax

**Published:** 1862-06

**Authors:** 


### PARACENTESIS IN HYDROTHORAX.

Trousseau frequently resorts to the use of paracentesis in cases of effusion into the chest, and has met with considerable success in this operation. During the past winter, he has had several cases of this nature, some of whom succumbed before the progress of the malady, while others recovered. He even ventures to puncture the pericardium. In a case of heart disease which he had a short time ago, he entertained the idea of performing the operation, but was fortunately, from some concomitant circumstances, prevented from carrying his intention into execution, for, upon making the post mortem examination, the amount of effusion was found to be very trifling. He has at present a case in the wards, of a boy whose chest was punctured for a pleuritic effusion, and who is now doing so well that in a few days he will be dismissed. The following is a short sketch of rather an interesting case, in which paracentesis thoracis was resorted to, but in this instance only as a palliative. A young woman, aged 22, was admitted on the 25th of February.

She had been delivered at the Maternity Hospital on the 14th of the same month, under the influence of chloroform, the labor having been severe and tedious. Eight days after her confinement, she returned home, but was compelled, on the above date, to seek admission into the Hotel Dieu, for a cold which she had caught in consequence of imprudent exposure. On admission, she appeared to be very anæmic, a bruit was audible in the vessels of the neck, both arterial and venous, and under the microscope the blood showed numerous isolated large white corpuscles. During the evening and night of this day, she experienced severe and prolonged rigors, with considerable oppresion of breathing, pulse weak and 108 per minute, tongue coated, with thirst, loss of appetite, and constipation. On the 28th, there was a repetition of the shivering, and she complained of pain in the right breast, which was much swollen, as was also the left; this was attributed to suppression of the lacteal secretion, her child having been sent to the depot, where it died of convulsions shortly afterwards. In the evening, she was suddenly seized with a most violent and excruciating pain in the right side, and at the same time, the respiration became very laborious. She was ordered sinapisms, hot fomentations, and applications of chloroform. On the 1st of March, the breast was still swollen and painful; the pulse 140 per minute, oppression of breathing considerable, and anxiety of countenance very marked. There was great pain on the slightest pressure being made on the thorax; there was also dulness at the lower part of the right side of the chest, with large mucous rales, but neither vesicular breathing nor ægophony was audible. On the 2d of March, at the upper part of the right chest, there was heard loud tympanitic resonance, with amphoric breathing and metallic succussion sound, but no vesicular murmur; at the lower part of the same side there was dulness with distant mucous rales; and the left side was normal. There was a very trifling expectoration of saffron-colored viscous phlegm; no history of a tuberculous tendency could be elicited. The diagnosis given was circumscribed pneumonia, and metastatic abscess from puerperal pyæmia, perforation of the lung, and, as a consequence, hydro-pneumo-thorax. Ordered subcutaneous injections of solution of atropine night and morning, with administration of mercury and digitalis. On the 3d of March, these symptoms continued; there was marked prominence of the upper part of the right chest anteriorly, and the respiration was entirely performed by the left side; gums slightly swollen. Ordered to suspend the calomel, to continue the digitalis, and to apply chloroform liniments. On the 4th, the patient's condition had become so alarming, from the extreme exhaustion and the oppression of breathing, that it was deemed necessary to perform paracentesis, which was done by entering a trocar between the seventh and eighth ribs, and drawing off a quantity of horribly fœtid, sero-purulent fluid. The proceeding afforded immense relief to the patient. A gum-elastic sound was retained in the aperture, in order to keep it permanent. On several subsequent occasions more of the same kind of fluid was withdrawn, and injections of a strong alcoholic solution of iodine and iodide of potassium were made, without producing much pain. Up to the 6th of March, four *litres* (French) had been withdrawn, ameliorating in a marked degree the condition of the patient. On the 7th, diarrhœa had set in, with great anxiety and oppression, the pulse was 158 per minute and very feeble, and in the chest a faint transmitted respiratory murmur was audible. The succession sound was diminished, but there was still great metallic tinkling posteriorly, and the thorax had commenced to contract. The iodine injections were continued, and chalk and bismuth ordered. After this the patient gradually sank, and expired on the 13th. The post mortem examination gave the following results:—perforation of the summit of the right lung, with circumscribed pneumonia and purulent deposit; the pleural cavity lined with a thick false membrane in a state of putrefaction; no tubercles in the lung; uterus healthy, except some deep engorgement of the os and cervex; no pus found in the cavity or structure of either uterus or Fallopian tubes.—*Paris Correspondence of the Edinburgh Medical Journal.*

